# Restrictive versus standard hyperhydration during high-dose cyclophosphamide for hematopoietic stem cell transplantation: a retrospective cohort study

**DOI:** 10.1007/s00520-026-10465-9

**Published:** 2026-02-23

**Authors:** R. J. Boosman, R. M. Chan, E. Nur, H. J. Huls, M. Crul, M. R. Heerma van Voss

**Affiliations:** 1 Department of Clinical Pharmacology and Pharmacy, Amsterdam UMC, De Boelelaan 1117, 1081 HV Amsterdam, the Netherlands; 2 Department of Pharmaceutical Sciences, Faculty of Science, Utrecht University, Leuvenlaan 4, 3584 CE Utrecht, the Netherlands; 3 Department of Hematology, Amsterdam UMC, De Boelelaan 1117, 1081 HV Amsterdam, the Netherlands; 4 Department of Blood Cell Research, Sanquin Research and Landsteiner Laboratory, Amsterdam, the Netherlands

**Keywords:** Hydration protocol, Hyperhydration, Cyclophosphamide, Hemorrhagic cystitis, Hematopoietic stem cell transplantation

## Abstract

**Purpose:**

Cyclophosphamide is a commonly used chemotherapeutic agent in hematopoietic stem cell transplantation (HSCT), but its use can lead to adverse effects such as hemorrhagic cystitis (HC) and electrolyte disturbances, including hyponatremia. While standard hydration protocols are used to mitigate these risks, the optimal regimen remains unclear. This study explores the impact of a restrictive hydration regimen on HC incidence and electrolyte imbalances in patients undergoing high-dose cyclophosphamide treatment as part of HSCT conditioning.

**Methods:**

A retrospective cohort study was conducted at Amsterdam UMC, including patients who received high-dose cyclophosphamide as part of HSCT between 2016 and 2024. Patients were grouped based on hydration protocols: an original regimen (5 L of NaCl 0.45%/dextrose 2.5% per day) and a new restrictive regimen (1.5 L/m^2^/day of 0.65% NaCl). The primary endpoint was the incidence of HC, while secondary endpoints included sodium and potassium changes, fluid overload (measured by furosemide use), and clinical outcomes.

**Results:**

HC occurred in 10/386 (2.6%) patients in the original protocol and 1/69 (1.4%) in the restrictive protocol (odds ratio [95% confidence interval]: 0.55 [0.03–2.96], *p* = 0.57). Clinically relevant hyponatremia was less common with the restrictive regimen (1.4%) than with the original protocol (4.4%), though the difference was not significant (*p* = 0.27). On the other hand, patients receiving the restrictive regimen showed more clinically relevant hypokalemia (8.7% vs 5.9%, *p* = 0.28). Fluid overload, as indicated by furosemide use, was lower in the restrictive group albeit not statistically significant.

**Conclusion:**

In this retrospective single-center cohort, we did not observe a higher incidence of HC or electrolyte imbalances with a restrictive hydration regimen compared to the original regimen.

## Introduction

Cyclophosphamide, a chemotherapeutic agent, is widely utilized in the treatment of various hematologic and oncologic conditions, including the conditioning regimen in hematopoietic stem cell transplantation (HSCT) [1]. The standard dosing regimen for cyclophosphamide in HSCT is dependent on both the specific type of transplant and individual patient characteristics, typically ranging from 50 to 60 mg/kg [1]. Cyclophosphamide is a prodrug that undergoes metabolism in the body, yielding both therapeutic and toxic metabolites, including the active phosphoramide mustard and the toxic metabolite acrolein. Accumulation of acrolein in the bladder is a key factor in the development of hemorrhagic cystitis (HC) [1–3]

Although cyclophosphamide-induced HC in HSCT recipients not receiving preventive measures is a major concern, the exact incidence remains inadequately documented, with reported rates varying between 4 and 36% [4]. To mitigate the risk of HC, preventive interventions such as 2-mercaptoethanesulfonic acid (Mesna), hyperhydration, and bladder irrigation are commonly used [5]. However, the optimal prophylactic strategy for HC remains uncertain, while some evidence showing inconsistency or even absence of effectiveness of these measures in some cases [6]. Moreover, the standard hyperhydration regimen, although beneficial in reducing HC risk, can cause fluid overload, potentially increasing the risk of cardiotoxicity [7–9].

In addition to HC, cyclophosphamide treatment in HSCT patients is also associated with an increased risk of hyponatremia. A small-scale study of 17 patients receiving ≥ 50 mg/kg of cyclophosphamide revealed that 82% of these patients experienced water intolerance, which led to a decrease in serum sodium levels [10]. The exact mechanism underlying this effect remains only partially understood, though potential factors include increased permeability of the renal distal tubules to water, increased release of antidiuretic hormone (ADH), and excessive fluid administration [11–13]. While data in adults are limited, it has been noted that intravenous isotonic fluids are safe and reduce the risk of hyponatremia by half compared to hypotonic fluids in pediatric patients [14, 15]. In contrast, the administration of hypotonic fluids has been associated with the development of hyponatremia. These findings suggest that hypotonic fluids, excessive fluid volumes, and ADH-mediated mechanisms contribute to the development of hyponatremia [16]. However, to date, there are no published data addressing the incidence of hyponatremia in adults undergoing cyclophosphamide treatment with different hydration regimens.

Given the high prevalence of cardiovascular complications among hematologic patients [17], the potential benefits of reducing the volume and amount of sodium in hyperhydration regimens during cyclophosphamide treatment remain unexplored. We hypothesized that a restrictive hydration protocol with higher sodium concentration would not increase HC incidence and would reduce fluid overload. This study seeks to investigate the impact of a reduced volume hyperhydration regimen on the development of hemorrhagic cystitis and electrolyte disturbances, such as hyponatremia, in order to better inform clinical practice and minimize associated toxicities.

## Materials and methods

### Study design and patients

This retrospective cohort study was conducted at Amsterdam UMC, Amsterdam, the Netherlands. This study was approved by the local institutional review board (study number: 2024.0125), which waived the need for acquiring additional informed consent because of the retrospective design of the study.

The study aimed to evaluate an adapted hydration protocol for patients with hematological malignancies undergoing high-dose cyclophosphamide as part of their hematopoietic stem cell therapy. In May 2023, the hospital’s hydration protocol transitioned from an original regimen to a restrictive protocol. The original hydration regimen for high-dose cyclophosphamide (> 1500 mg/m^2^/day or ≥ 40 mg/kg/day), administered with Mesna, involved the administration of over 5 L of NaCl 0.45%/dextrose 2.5% daily until 24 h after the last cyclophosphamide administration. The restrictive protocol involved 1.5 L/m^2^/day of 0.65% NaCl until 24 h after the last cyclophosphamide administration. The administration of Mesna remained unchanged at 20 mg/kg four times daily, while the potassium dosage was altered from 100 to 60 mmol/day under the restrictive protocol.

All adult (≥ 18 years) patients receiving high-dose cyclophosphamide as part of a first HSCT therapy between April 2016 and April 2024 were eligible for inclusion in this study. The study population was categorized into two cohorts based on the hydration protocol administered. Patients were excluded if they did not receive the hydration protocol under investigation or if data were missing.

### Data collection

Data were collected from the electronic patient records (EPIC, EPIC Systems, Verona, WI, USA). Patient characteristics, including demographic data (age, biological gender, and weight), renal function, cyclophosphamide dosage, type of hydration regimen, furosemide dose (as an indicator of fluid overload), and laboratory serum markers (creatinine, sodium, and potassium) were recorded at baseline and during the first week following high-dose cyclophosphamide administration. The incidence of HC was assessed based on the treating physician’s notes, in addition to the use of a catheter during hydration. Data for all parameters were collected from one week before to one week after hydration.

### Endpoints

The primary endpoint was the difference in incidence of HC with the restrictive protocol compared to the original protocol. HC was diagnosed if it was noted by the physician as the most likely diagnosis or if hematuria occurred with platelet levels > 20 × 10^9^/L, in the absence of alternative explanations (e.g., BK-virus or urinary tract infection) within 7 days following high-dose cyclophosphamide administration. The use of a catheter during cyclophosphamide administration was recorded as catheter use.

Secondary endpoints included the change in sodium and potassium levels. The change in sodium levels was analyzed by comparing the difference between baseline and the lowest sodium level recorded after hydration for each patient (denoted as delta sodium) and by examining the overall differences in outcomes between hydration groups. The influence of hydration on potassium levels was assessed by comparing baseline and post-hydration potassium levels, accounting for potassium supplementation when necessary. Additionally, the change in sodium and potassium levels was scored according to the Common Terminology Criteria for Adverse Events (CTCAE) version 5.0 criteria for hyponatremia and hypokalemia, respectively [18].

Fluid overload was evaluated by comparing cumulative furosemide use up to 1 week after hydration between hydration regimens and by analyzing the distribution of furosemide doses within each group. Oral furosemide doses were adjusted using a mean bioavailability of 45% [19].

### Statistical analysis

Statistical differences between patient characteristics in the two groups were assessed using chi-squared tests, Fisher’s exact tests, or *t*-tests, as appropriate, based on normality assessed via the Shapiro–Wilk test. A *p*-value of < 0.05 was considered statistically significant, and Bonferroni correction was applied to account for multiple comparisons. To assess the effect of the adapted hydration protocol on individual covariates, a univariate regression analysis was performed. The type of regression analysis (linear, logistical, ordinal, or quantile regression analysis) was based on the type and normality of the (log-transformed) data. For the primary endpoint (HC), a Firth’s penalized logistic regression analysis was performed adjusting for the type of treatment protocol, the cyclophosphamide dose, the baseline eGFR, and the year of administration (to account for secular trends). All statistical analyses were performed using R (version 4.5.0, R-project, Vienna, Austria).

## Results

**Fig. 1:**
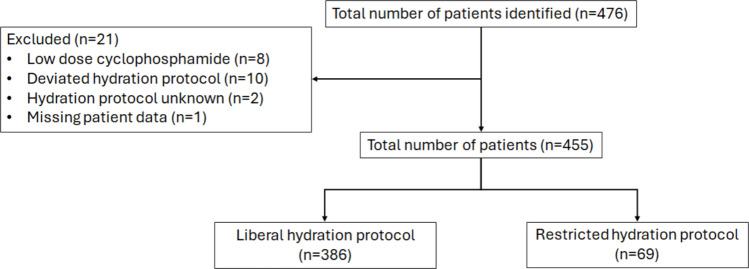
Flowchart of the included patients

Out of a total of 476 identified patients, 455 were eligible for inclusion in this study, of which 386 received the original hydration protocol and 69 the restrictive hydration protocol. Figure 1 illustrates the flowchart of the study participants.

The baseline characteristics of patients are presented in Table 1. Bonferroni correction led to a significance level of 0.0042. In general, the baseline demographics were comparable between the two groups. However, patients receiving the original hydration protocol were administered a higher body-weight-based dose of cyclophosphamide. Additionally, there was a statistically significant difference in the treatment protocols between the two hydration groups, with notable variations in the use of protocol 1 (busulfan and cyclophosphamide (60 mg/kg) prior to an autologous HSCT) and protocol 2 (reduced intensity scheme with cyclophosphamide (50 mg/kg), fludarabine, and total body irradiation). Therefore, a subgroup analysis was also performed for patients receiving a cyclophosphamide dose of 50 mg/kg. Within this subgroup analysis, no significant differences were observed between the two groups.

**Table 1:** Baseline characteristics of patients in both the total patient group and subgroup cohort. Bonferroni-corrected *p*-values beneath the significance level of 0.0042 (for the total cohort) and 0.0045 (for the subgroup analysis) are bolded. Values are presented as number (percentages) or as median [interquartile range] as appropriate

Patient demographic	Total patient cohort			50 mg/kg subgroup cohort		
	5 L/day of 0.45% NaCl/ 2.5% dextrose	1.5 L/m^2^/day of 0.65% NaCl	*p*-value	5 L/day of 0.45% NaCl/ 2.5% dextrose	1.5 L/m^2^/day of 0.65% NaCl	*p*-value
	(*N* = 386)	(*N* = 69)		(*n* = 239)	(*n* = 65)	
**Gender, male**	234 (60.6)	41 (59.4)	0.96	151 (63.2)	39 (60.0)	0.75
**Age in years, median**	58 [44–66]	60 [45–66]	0.46	60 [45–66]	60 [46–66]	0.85
**Weight (kg)**	80 [68–89]	76 [67–90]	0.34	81 [69–90]	77 [67–90]	0.23
**Baseline eGFR (mL/min)**			0.81			0.75
≥ 90	251 (65.1)	45 (65.2)		156 (65.3)	41 (63.1)	
60–89	110 (28.5)	19 (27.5)		69 (28.9)	19 (29.2)	
30–59	18 (4.7)	5 (7.2)		11 (4.6)	5 (7.7)	
< 30	1 (0.3)	0		1 (0.4)	0	
**Baseline sodium (mmol/L)**	138 [136–140]	138 [135–139]	0.03	138 [136–140]	137 [135–139]	0.33
**Baseline potassium (mmol/L)**	4.0 [3.7–4.2]	4.0 [3.7–4.2]	0.14	3.9 [3.7–4.1]	4.0 [3.7–4.2]	0.01
**Cyclophosphamide dosage (mg/kg)**						
40	14 (3.6)	1 (1.4)	**9.5 × 10**^**−7**^	0	0	**-**
50	239 (61.9)	65 (94.2)		239 (100)	65 (100)	
60	133 (34.4)	3 (4.3)		0	0	
**Total cyclophosphamide (mg)**	8400 [7130–9660]	6680 [7760–9000]	0.008	8120 [6920–9000]	7720 [6680–8960]	0.23
**Catheter, yes**	25 (6.5)	4 (5.8)	1	13 (5.4)	4 (6.2)	1
**Treatment protocol**			**5.4 × 10**^**−5**^			0.57
ATG/thiotepa/Flu/Cy/TBI/PTCY (NMA)	8 (2.1)	2 (2.9)		8 (3.3)	2 (3.0)	
Bu4/Cy auto HSCT (MA)	89 (23.1)	1 (1.4)		0	0	
Cy/flu/TBI/PTCY (NMA)	144 (37.3)	37 (53.6)		144 (60.3)	37 (56.9)	
Cy/TBI (MA)	13 (3.4)	0		0	0	
FLAMSA-RIC/PTCY (NMA)	5 (1.2)	3 (4.3)		5 (2.1)	3 (4.6)	
FLAMSA-RIC (NMA)	45 (11.7)	3 (4.3)		0	0	
Bu3/Flu/PTCY (MA)	60 (4.7)	15 (21.7)		60 (2.5)	15 (2.3)	
Alemtuzumab/Flu/Mel/PTCY (NMA)	4 (1.0)	0		4 (1.7)	0	
TBI/PTCY (MA)	18 (4.7)	8 (11.6)		18 (7.5)	8 (12.3)	
Total hydration content*						
Fluid (L)	10	5.8 [5.4–6.2]	** < 1.0 × 10**^**−16**^	10	5.8 [5.4–6.3]	** < 1.0 × 10**^**−16**^
Sodium (mmol)	770	647 [600–694]	** < 1.0 × 10**^**−16**^	770	647 [593–697]	** < 1.0 × 10**^**−16**^
Potassium (mmol)	200	120	** < 1.0 × 10**^**−16**^	200	120	** < 1.0 × 10**^**−16**^

*ATG*, anti-thymocyte globulin; *Bu3*, busulphan 3-day schedule; *Bu4*, busulphan 4-day schedule; *Cy*, cyclophosphamide; *eGFR*, estimated glomerular filtration rate; *FLAMSA-RIC*, fludarabine/cytarabine/amsacrine-reduced intensity concept; *Mel*, melphalan; *Flu*, fludarabine; *HSCT*, hematopoietic stem cell transplantation; *MA*, myeloablative; *NMA*, non-myeloablative; *PTCY*, post-transplantation cyclophosphamide; *TBI*, total body irradiation

*Total content received during the 2-day hydration regime

### Primary outcome

Table 2 outlines the principal outcomes. The incidence of HC following cyclophosphamide therapy did not differ significantly between hydration protocols in the total cohort (OR (95% CI): 0.55 (0.03–2.96), *p* = 0.57) or in the subgroup cohort (OR (95% CI): 0.73 (0.04–4.64), *p* = 0.77). In multivariable logistic regression using Firth’s penalized likelihood, adjusted for treatment protocol, prescribed cyclophosphamide dose, eGFR, and year of treatment, the adjusted odds ratio for hydration protocol was 1.44 (95% CI: 0.003–10.8). Other covariates also showed no significant association with HC risk: treatment protocol 0.99 (0.49–1.55), cyclophosphamide dose 0.99 (0.76–1.66), baseline eGFR 1.01 (0.91–1.42), and year of administration 0.94 (0.73–1.20).

**Table 2:** Comparison of outcomes between hydration regimens: univariate regression analyses for the primary and secondary objective(s) for both the total patient cohort and the subgroup cohort. Values are presented as number (percentages) or as median [interquartile range] as appropriate. Statistical significant outcomes are bolded

Outcome and measurements	Total patient cohort			50 mg/kg subgroup cohort		
	5L/day of 0.45% NaCl/ 2.5% dextrose	1.5L/m^2^/day of 0.65% NaCl	*p*-value	5L/day of 0.45% NaCl/ 2.5% dextrose	1.5L/m^2^/day of 0.65% NaCl	*p*-value
	(*N* = 386)	(*N* = 69)		(*n* = 239)	(*n* = 65)	
**Hemorrhagic cystitis, yes**	10 (2.6)	1 (1.4)	0.57	5 (2.1)	1 (1.5)	0.77
**Lowest post-chemo sodium (mmol/L)**	135 [131–137]	134 [130–137]	0.28	134 [131–137]	134 [130–136]	1.00
**Delta sodium (mmol/L)**	−3 [−7 to −1]	−4 [−6 to −2]	0.20	−3 [−7 to −1]	−4 [−6 to −2]	0.14
**CTCAE term hyponatremia**			0.55			0.88
Grade 0 (≥ 135 mmol/L)	194 (50.3)	32 (46.4)		111 (46.4)	29 (44.6)	
Grade 1 (130–134 mmol/L)	135 (35.0)	25 (36.2)		88 (36.8)	25 (38.5)	
Grade 2 (125–129 mmol/L)	40 (10.4)	11 (15.9)		29 (12.1)	10 (15.4)	
Grade 3* (120–124 mmol/L)	12 (3.1)	0		8 (3.3)	0	
Grade 4* (< 120 mmol/L)	5 (1.3)	1 (1.4)		3 (3.3)	1 (1.5)	
Delta grade hyponatremia	0 [0–1]	0 [0–1]	0.58	0 [0–1]	1 [0–1]	0.57
**Post-chemo potassium (mmol/L)**	3.7 [3.4–4.0]	3.7 [3.3–3.9]	1.00	3.8 [3.5–4.1]	3.8 [3.3–4.0]	1.00
**Delta potassium (mmol/L)**	−0.2 [−0.5 to 0.1]	−0.3 [−0.6 to −0.1]	0.16	−0.1 [−0.3 to 0.2]	−0.3 [−0.5 to −0.1]	**0.008**
**CTCAE grade hypokalemia**						
Grade 0 (≥ 3.5 mmol/L)	285 (73.8)	46 (66.7)	0.21	192 (80.3)	46 (70.8)	0.06
Grade 1/2 (3–3.5 mmol/L)	78 (20.2)	17 (24.6)		42 (17.6)	13 (20.0)	
Grade 3* (2.5–3 mmol/L)	19 (4.9)	6 (8.7)		3 (1.3)	6 (9.2)	
Grade 4* (< 2.5 mmol/L)	4 (1.0)	0		2 (0.8)	0	
Delta grade hypokalemia	0 [0–1]	0 [0–1]	0.60	0 [0–0]	0 [0–0]	0.11
Furosemide dose (mg)	40 [20–80]	40 [20–60]	1.00	50 [20–80]	40 [20–60]	0.31

*CTCAE grade ≥ 3 is considered clinical relevant

### Secondary outcomes

The lowest post-chemotherapy sodium levels were comparable between the 0.45% NaCl/2.5% dextrose and 0.65% NaCl regimens. In the total patient cohort, the median sodium was 135 mmol/L and 134 mmol/L, respectively (*p* = 0.28), while in the subgroup analysis, it was 134 mmol/L and 134 mmol/L (*p* = 1.00). There were also no statistically significant differences in delta sodium or CTCAE grading for hyponatremia. The incidence of clinically relevant hyponatremia (defined as ≥ grade 3) did not differ significantly in either the total cohort (OR: 0.32 (0.02–1.59), *p* = 0.27) or the subgroup analysis (OR: 0.32 (0.02–1.71), *p* = 0.29).

Potassium levels following chemotherapy did not differ significantly between the hydration groups in the overall patient cohort. However, subgroup analysis revealed a statistically significant smaller decrease in potassium levels among patients receiving the original hydration protocol compared to those receiving the restrictive protocol (mean change in potassium: −0.1 mmol/L vs −0.3 mmol/L, *p* = 0.008). Despite reaching statistical significance, this difference is not considered clinically meaningful. While grade 4 hypokalemia was observed exclusively in the original hydration group, a higher incidence of clinically relevant (≥ grade 3) hypokalemia was noted among patients in the restrictive hydration group (OR: 4.76 (1.39–17.0), *p* = 0.012). Nonetheless, no statistically significant difference in clinically relevant hypokalemia was observed when analyzing the entire cohort (OR: 1.12 (0.91–1.39), *p* = 0.28).

Furosemide use was similar across both groups. In the subgroup analysis, the median furosemide dose was 40 mg for both regimens (*p* = 0.64). In the total patient group, it was 50 mg for the 0.45% NaCl/2.5% dextrose group and 40 mg for the 0.65% NaCl group (*p* = 0.31). Overall, although not statistically significant, a higher percentage of patients in the original hydration group received a furosemide dose ≥ 250 mg when compared to the restrictive hydration regimen (5.2% vs 1.4%, *p* = 0.75).

## Discussion

This study presents novel insights into the impact of hydration protocols on the incidence of HC and electrolyte management in patients receiving high-dose cyclophosphamide as part of the conditioning regimen for hematopoietic stem cell transplantation. We did not find evidence that a more restrictive hydration regimen is associated with an increased incidence of HC or electrolyte imbalances compared to a liberal hydration regimen. These results have significant clinical implications, particularly given the growing interest in optimizing supportive care for hematologic malignancy patients undergoing intensive chemotherapy.

The incidence of HC in the restrictive group was not higher than in the original hydration group. The absolute difference between the two regimens was small. This suggests that factors beyond hydration volume, such as Mesna administration and catheter use, may be more influential in determining the risk of HC. Notably, both regimens maintained equivalent levels of Mesna and catheter use, mitigating the potential confounding effects of these variables on HC outcomes.

Our study is the first to directly assess the effect of reduced hydration volume on HC incidence in this patient population. Previous research demonstrated that both Mesna and extensive hydration are effective in reducing HC incidence [7–9], while it was postulated that Mesna could reduce the need for hyperhydration without increasing the risk of HC [20, 21]. In line with these findings, our results indicate that a daily fluid intake of approximately 3 L might be as effective as 5 L in preventing HC, without any apparent adverse effect. These findings contribute to the growing body of evidence supporting the potential to optimize hydration regimens without compromising prophylactic efficacy.

Regarding sodium levels, the results deviated from our hypothesis. We expected that less hypotonic would result in higher sodium levels, but this was not observed. Van Regenmortel et al. previously demonstrated that solutions with higher sodium content lead to increased sodium load, a key rationale for transitioning to a restrictive hydration regimen to prevent hyponatremia and fluid overload [22]. While we found no significant differences in sodium levels between the two groups overall, the restrictive regimen was associated with better management of clinically relevant (≥ grade 3) hyponatremia in both the total patient group and the subgroup cohort (4.4–6.6% vs 1.4–1.5%, respectively). This might suggest that a reduced fluid volume may mitigate the risk of severe hyponatremia, which has been described earlier [23].

A statistically significant reduction in potassium levels was observed in patients receiving the restrictive hydration regimen. This finding is consistent with the lower potassium content administered in this group. Furthermore, the incidence of clinically relevant hypokalemia (≥ grade 3) was higher among patients in the restrictive protocol compared to those receiving the original hydration protocol (8.7–9.2% vs 2.1–5.9%, respectively). In the subgroup analysis, this difference reached statistical significance. These findings prompt important considerations regarding the adequacy of current potassium supplementation within the restrictive hydration protocol and highlight the need for further investigation into the complex interplay between hydration volume, electrolyte management, and associated complications in this high-risk patient population.

The original hydration regimen, with its larger volume of fluid and higher sodium chloride content, was associated with a higher incidence of clinically relevant fluid overload, as evidenced by increased furosemide use (> 250 mg). This finding aligns with previous research suggesting that excessive fluid administration can lead to more severe fluid overload [20].

This study offers valuable insights into the effects of hydration regimens on clinical outcomes. However, several limitations should be acknowledged. First, the small sample size in the restrictive hydration regimen group limits the statistical power of the study. A larger cohort would allow for the detection of more subtle differences in clinical outcomes and provide more robust data on subgroups. Additionally, the retrospective nature of the study introduces potential bias, including inaccuracies in HC diagnosis based on physician notes in the hospital information system. The assessment of HC may have been influenced by unconfirmed diagnoses or overlapping conditions such as (asymptomatic) BK polyomavirus infections, which could have confounded the results. Furthermore, the study did not account for additional fluid intake outside the prescribed hydration regimens, which could have introduced variability between groups. Other potential confounders, such as the influence of concomitant medications on electrolyte levels or calendar-time differences in monitoring, supportive care, or other treatment strategies changing over time, were not considered, which may have affected the results. Finally, the study’s reliance on the lowest sodium levels as a marker for hyponatremia may have led to underreporting of grade 3 hyponatremia, as symptomatic grade 2 hyponatremia may have been misclassified.

This study adds to the limited evidence on the effects of hydration regimens on HC and electrolyte management in adults, providing valuable insights into how varying hydration volumes and NaCl concentrations influence clinical outcomes. We did not identify a significant difference between an original and restrictive hydration regimen with regard to HC incidence and electrolyte disorders, suggesting that restrictive hydration regimens can safely be applied. However, since this study is relatively small, future studies with larger sample sizes and sufficient power are needed to validate our findings. Additionally, investigating the effects of NaCl 0.9% as a hyperhydration fluid may yield further insights.

In conclusion, a more restrictive hydration regimen was not associated with an increased incidence of HC and may offer improved management of electrolyte imbalances and fluid overload. These findings suggest that the restrictive hydration protocol is a viable alternative to the original regimen. However, further prospective studies with larger cohorts and more comprehensive data collection are needed to confirm these results and refine the optimal hydration strategy for patients undergoing high-dose cyclophosphamide therapy.

## Data Availability

Data are available upon reasonable request and according to the institutional data sharing policy by sending an e-mail to the corresponding author.
